# Guillain-Barre Syndrome, Neuroborreliosis, or Both

**DOI:** 10.7759/cureus.7823

**Published:** 2020-04-25

**Authors:** Joey Owens, Asia Filatov, Sameea Husain-Wilson

**Affiliations:** 1 Internal Medicine, Charles E. Schmidt College of Medicine, Florida Atlantic University, Boca Raton, USA; 2 Neurology, Charles E. Schmidt College of Medicine, Florida Atlantic University, Boca Raton, USA; 3 Neurology, Marcus Neuroscience Institute, Boca Raton Regional Hospital, Boca Raton, USA

**Keywords:** guillain-barré syndrome (gbs), lyme disease, neuroborreliosis, borrelia burgdorferi, campylobacter jejuni, facial nerve palsy, tick

## Abstract

Guillain-Barre syndrome (GBS) is an acute paralytic neuropathy. Limited reports of GBS caused by tick-borne pathogens exist. Lyme disease is a tick-borne infectious disease that is commonly caused by Borrelia burgdorferi. The nervous system may be involved and is called neuroborreliosis.

In this case, we report a 30-year-old female who presented to the emergency department with one week of diffuse, increasing weakness in the upper/lower extremities and face after a recent gastrointestinal illness and travel to the Northeastern United States. Areflexia was noted in bilateral lower extremities. Lumbar puncture results together with clinical presentation were consistent with a diagnosis of GBS. Lab results later revealed a possible Lyme infection in cerebrospinal fluid, which along with recent travel to endemic area gave high suspicion for Lyme disease. The patient was treated for both diseases with significant improvement.

Taking a good history is an essential first step in diagnosis, as travel history was key in testing for Lyme.

## Introduction

Guillain-Barre syndrome (GBS) affects about 100,000 people per year worldwide. GBS is usually preceded by an infection or other event that stimulates the immune system and causes an autoimmune reaction that targets the peripheral nerves. The most common antecedent event is a gastrointestinal infection (GI) due to Campylobacter jejuni [1]. Other causes that have been documented include cytomegalovirus, Epstein-Barr virus, human immunodeficiency virus (HIV), and more recently, Zika virus [2]. There have been limited reports of GBS caused by tick-borne pathogens such as Borrelia burgdorferi [3-6]. The typical presentation of GBS is an acute progression of limb weakness one to two weeks after immune stimulation with a peak in the weakness at about two to four weeks [7]. Beyond the nerves of the peripheral limbs, the facial nerve is also commonly involved [1]. Treatment of the condition consists of either administration of intravenous immunoglobulin (IVIG) or plasmapheresis. Neither treatment has been found to be superior to the other, and the decision between the two is based on local resources and preference.

The progression of Lyme disease has three clinical stages: early localized, early disseminated, and late disease. The nervous system may be involved in the disseminated state and is called neuroborreliosis which is only seen in about 10%-15% of cases of Lyme disease in the United States. There is a classic clinical triad that occurs in neuroborreliosis, which includes lymphocytic meningitis, cranial neuritis, and radiculoneuritis. Diagnosis is made by having a possible exposure to ticks, supportive clinical features, and positive serology and/or antibodies in the cerebrospinal fluid (CSF).

This case report examines a patient who presented to the emergency department (ED) with an increasing peripheral weakness including facial nerve involvement after a recent GI illness and travel to an area of the United States where tick-borne pathogens are endemic.

## Case presentation

A 30-year-old female with a past medical history significant for total thyroidectomy on levothyroxine developed a sinus infection, cold-like symptoms, and a severe headache so she decided to see an outpatient neurologist. She had a brain MRI which was normal. She was told she was having a complex migraine and was not prescribed any medications. She then developed severe nausea and emesis, and therefore went to the ED for evaluation. She was ultimately told that she had gastroenteritis and was discharged home from the ED. One week later, she began to experience muscle weakness which began in the upper and lower extremities but progressed to her facial muscles. She again presented to the ED due to the increasing severity of the weakness. In the ED she was unable to ambulate, get out of bed, or speak more than a few words at a time. The cold-like symptoms, nausea, and vomiting had subsided by this time, but she still had headache and had developed right-sided jaw pain, tongue swelling, very mild neck pain, and paresthesias in her toes and fingers. She revealed in the ED that two weeks ago she had returned home to Florida from a trip to North Carolina where she spent time in a suburban setting. She did not go camping or spend any time in a rural setting. She did not notice any tick bites, although she admits to having one to two mosquito bites.

**Figure 1:**
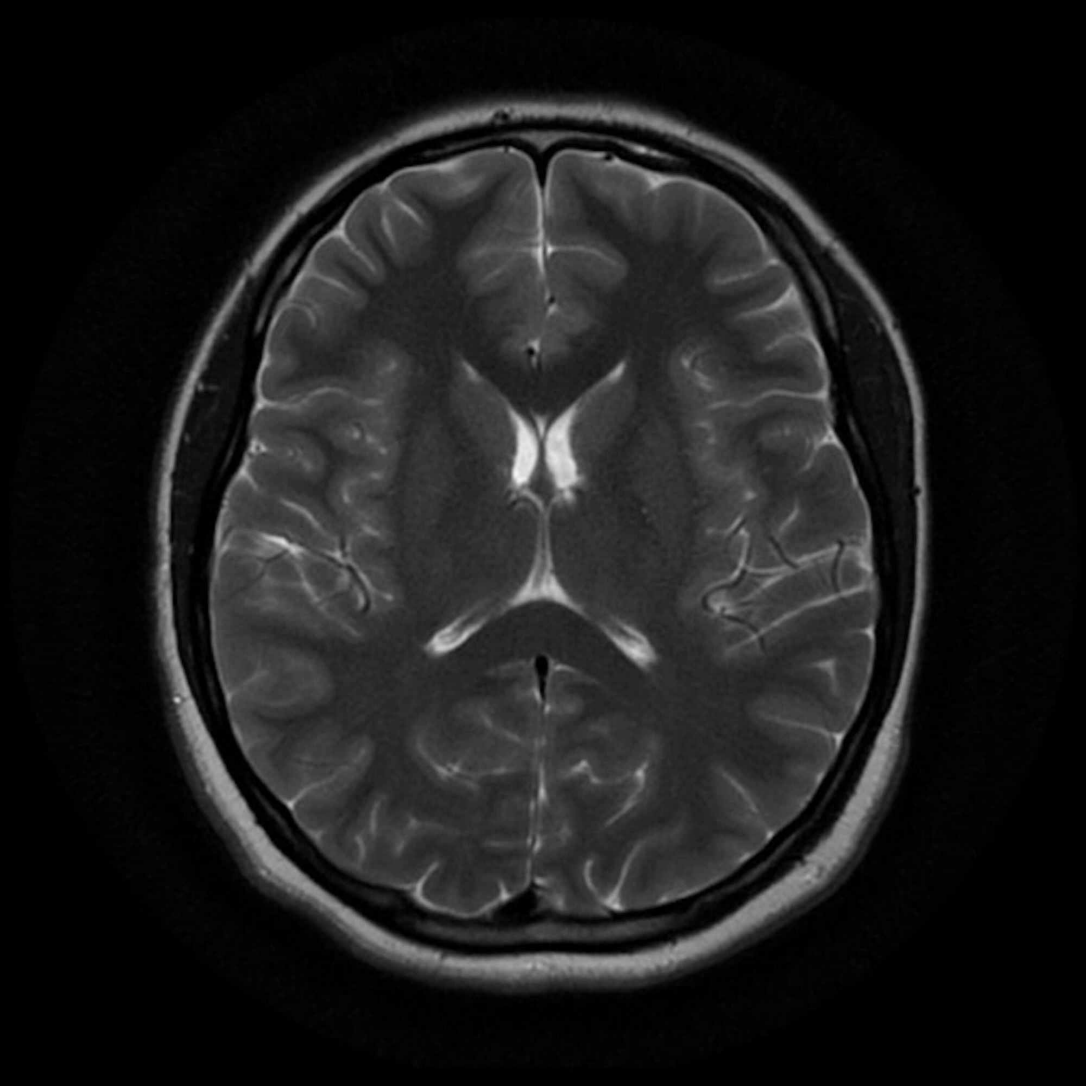
Normal Brain MRI

Neurology was consulted due to the weakness, headache, paresthesias, and jaw pain. In the ED she was afebrile without leukocytosis. On physical exam, she had symmetric 4/5 strength in the upper extremities and symmetric 2/5 strength in the lower extremities. She was hyporeflexic in the upper extremities and areflexic in the lower extremities. She was admitted to the hospital, and her initial workup included a complete blood count, basic metabolic profile, chest X-ray, and electrocardiogram which were all unremarkable. Additional blood work was ordered including thyroid-stimulating hormone, rapid plasma reagin, hepatitis C, HIV, vitamin B1, B6, D, E, B12, and folate which were also all unremarkable. An MRI of the brain with and without contrast was ordered and was negative for meningeal enhancement (Figure 1). An magnetic resonance angiography (MRA) head without contrast and an MRA neck with and without contrast were ordered and were negative for vertebrobasilar insufficiency and carotid stenosis. After preliminary testing was deemed unrevealing, a lumbar puncture was performed.

CSF studies showed an elevated protein level of 298 mg/dL (range 15-45 mg/L), white blood cell (WBC) count of 6/mcL (range 0-4/mcL), and glucose of 56 mg/dL (range 40-75 mg/dL) (Table 1). CSF infectious screen for syphilis, cytomegalovirus, and Epstein-Barr virus were all negative. The CSF study values were indicative of albuminocytologic disassociation, which along with the clinical presentation were consistent with a diagnosis of GBS. Electromyography (EMG)/nerve conduction velocity (NCV) studies were not available in the inpatient setting. Treatment with a two-day course of IVIG 1 g/day was initiated along with a corticosteroid regimen consisting of an initial 20 mg dose with a three-week taper of 8 mg BID, 4 mg BID, and 2 mg BID. The patient had significant motor improvement after IVIG treatment. 

**Table 1:** Cerebrospinal Fluid Analysis

	Lumbar Puncture	Reference Values
Protein	298 mg/dL	15-45 mg/dL
Glucose	56 mg/dL	40-75 mg/dL
White Blood Cell	6/mcL	0-4/mcL

After treatment with IVIG had concluded, polymerase chain reaction results from the CSF became available indicating a possible Borrelia burgdorferi infection in the CSF, 1.20 Lyme Index Value (normal high ≤0.99). This lab finding along with the recent history of travel to the Northeastern United States was highly suspicious for Lyme disease. Infectious disease was consulted. At this point, the patient had not regained full recovery of motor function and thus treatment for Lyme disease was initiated. The patient was given a 14-day course of ceftriaxone 2 g/day. Two days after initiation of ceftriaxone, the patient was discharged home with a peripherally inserted central catheter line to continue the antibiotic treatment. At the time of discharge, the patient was able to ambulate on her own, with only minor residual weakness in all limbs. She had regained her ability to speak and had only mild paresthesias in her lips. She was given instructions to continue physical therapy, follow up with infectious disease, and follow up with neurology for outpatient EMG/NCV. 

## Discussion

The clinical case presentation and studies pointed towards GBS superimposed with Lyme disease caused by Borrelia burgdorferi. Typically, the limb weakness associated with GBS is preceded by GI illness with diarrhea. Our patient had nausea and emesis without diarrhea followed by limb weakness.

There is a possibility that the patient could have been exposed to an infected deer tick, Ixodes scapularis, which is a renowned vector of Borrelia burgdorferi. She recently visited an area endemic for Lyme disease. Lyme disease is endemic in the mid-Atlantic, Northeast, upper Midwest, and the Pacific Coast of the United States. The disease is common in the District of Columbia, Kentucky, North Carolina, Indiana, Illinois, Iowa, North Dakota, Michigan, South Dakota, West Virginia, Tennessee, and Ohio [8]. The reported mosquito bite does not lend itself useful in this case as the clinical presentation of the patient did not point towards any potential mosquito-transmitted infection.

The observed signs and symptoms were consistent with the clinical presentation of Lyme disease. In addition to suspected involvement of the glossopharyngeal nerve upon initial examination, there was weakness of the facial muscles with the inability to speak [1,9]. This is characteristic of the radiculoneuritis of the facial nerve seen in Lyme disease. Central nervous system involvement in Lyme disease may result in cranial neuritis, meningitis, radiculoneuritis or a combination of the syndromes [9,10]. MRI scanning did not indicate any meningeal involvement; however, the patient’s clinical exam was positive for both cranial neuritis and radiculoneuritis due to the paresthesias and speech difficulty. Our patient had an elevated protein level in the CSF which was consistent with an increased production of Borrelia burgdorferi immunoglobulin M antibodies in response to the infection. Lastly, Borrelia burgdorferi infection presents with an elevation of WBCs and glucose concentration in the CSF [9].

Ceftriaxone therapy typically results in marked improvement of the facial palsy and resolution of the ophthalmoplegia seen in Lyme disease [9]. Use of corticosteroids in the management of Lyme disease was contraindicated in this case since there was a long-term risk of facial palsy [11].

## Conclusions

GBS proved difficult to diagnose since it is a rare disease. History taking was vital in helping the healthcare team to make the correct diagnosis. The patient’s travel history was paramount in determining which studies to order. The disease showed improved treatment outcomes with the use of IVIG, steroids, and ceftriaxone.
